# *CDKN2A* Gene Expression as a Potential Aging Biomarker in Dogs

**DOI:** 10.3389/fvets.2021.660435

**Published:** 2021-04-26

**Authors:** Sára Sándor, Kitti Tátrai, Kálmán Czeibert, Balázs Egyed, Enikő Kubinyi

**Affiliations:** ^1^Department of Ethology, Senior Family Dog Project, ELTE Eötvös Loránd University, Budapest, Hungary; ^2^Department of Genetics, ELTE Eötvös Loránd University, Budapest, Hungary

**Keywords:** *CDKN2A*, dog, aging, biomarker, gene expression, blood

## Abstract

Describing evolutionary conserved physiological or molecular patterns, which can reliably mark the age of both model organisms and humans or predict the onset of age-related pathologies has become a priority in aging research. The age-related gene-expression changes of the *Cyclin Dependent Kinase Inhibitor 2A* (*CDKN2A*) gene have been well-documented in humans and rodents. However, data is lacking from other relevant species, including dogs. Therefore, we quantified the *CDKN2A* mRNA abundance in dogs of different ages, in four tissue types: the frontal cortex of the brain, temporal muscle, skin, and blood. We found a significant, positive correlation between *CDKN2A* relative expression values and age in the brain, muscle, and blood; however, no correlation was detected in the skin. The strongest correlation was detected in the brain tissue (*CDKN2A/GAPDH*: *r* = 0.757, *p* < 0.001), similarly to human findings, while the muscle and blood showed weaker, but significant correlation. Our results suggest that *CDKN2A* might be a potential blood-borne biomarker of aging in dogs, although the validation and optimization will require further, more focused research. Our current results also clearly demonstrate that the role of *CDKN2A* in aging is conserved in dogs, regarding both tissue specificity and a pivotal role of *CDKN2A* in brain aging.

## Introduction

Dogs have received growing attention as model animals of human aging and neurodegeneration because they are prone to developing similar age-related pathologies within a much shorter lifetime, and they are exposed to the same environmental and lifestyle stressors present in the modern human environment (1–4). Understanding how they age could also bestow benefits on the animals and their owners through advancing veterinary diagnostics.

Currently, in humans, the lack of universally applicable and highly reliable biomarkers, which can predict mortality and the onset of age-related pathologies is a major obstacle in the way of translational research goals and developing effective preventive approaches and therapies (5).

So far, several physiological and molecular parameters have been proposed as predictors of biological age and disease status in humans. However, most of these have limitations regarding clinical applicability and range of validity. For example, traditional clinical biomarkers, like blood pressure or cholesterol levels, which could predict mortality in younger cohorts, often lose their predictive capacity in older people (6, 7).

Epigenetic markers are promising candidates for developing a reliable and universal system predicting the course of aging (8, 9). Importantly, epigenetic signatures of certain genomic regions are conserved between humans, rodents, and dogs (10, 11) and an epigenome-based age-determination system has been recently developed for Labrador retriever dogs (12). Nevertheless, defining molecular markers beyond epigenetic patterns or telomere shortening has been recommended by several authors to increase the precision of predictions and to allow the monitoring of specific diseases (13, 14). Recently, for example, a panel of blood-borne markers was proposed as appropriate for geroscientific research in humans (15).

In dogs, the neuronal cytoskeletal protein neurofilament light chain (NfL) has recently been validated as a marker of age-related neurodegeneration, which could augment translational research as well (16). Gene expression based markers in general, however, may show fundamental differences between species as was reported by Hudgins et al. in 2018, when they compared the expression patterns in different tissues of mice and humans. In contrast, the expression of the p16(*INK4A)* transcript, together with p14(*ARF*) (p19 in mice), both encoded by the *Cyclin Dependent Kinase Inhibitor 2A (CKDN2A)* gene, have been reported to show a similar pattern of increased expression with aging in humans and rodents (17–19). The expression of the *CDKN2A* gene - which plays an important role in cell-cycle inhibition and is a tumor suppressor (20), has been reported to be an evolutionarily conserved regulator of cellular senescence (21). Its potential as a biomarker to predict biological age was proposed to even surpass telomeres in humans (22, 23). Also, it was shown to predict renal allograft function with higher accuracy than telomere length and chronological age (24).

Based on these results, *CDKN2A* expression has been proposed as a highly promising molecular biomarker of human aging (25–27). Furthermore, *CDKN2A* levels in blood were reported to be indicative of accelerated cellular aging caused by various infections or type 2 diabetes (28–30). Importantly, similar findings were reported in rodents [reviewed by Sorrentino et al. (31) in 2014], supporting the conserved nature of *CDKN2A* expression as a marker of cellular senescence. However, *CDKN2A*'s role in aging has not yet been investigated in other mammalian clades. Assessing the applicability of *CDKN2A* as an aging biomarker in dogs may further support reliable age-determination in translational research with this species (32). To this end, we investigated the mRNA expression of the canine *CDKN2A* gene in the brain (frontal cortex), muscle (m. temporalis), and skin (head area), obtained from pet dogs of various breeds within the frames of study 1, to test whether *CDKN2A* shows a similar tissue-expression pattern in this species as has been reported in humans and rodents. Due to methodological limitations, blood samples could not be analyzed from the same individuals (see details in Materials and Methods). Therefore, a population consisting of border collie dogs, which were available as participants of an independent study, were included in study 2 to assess the correlation between *CDKN2A* expression and age in blood.

## Methods

### Ethics Statement

The biological sample collection conducted in this study complied with national [Hungarian law (“1998. évi XXVIII. Törvény” 3. §/9.—The Animal Protection Act)] and EU legislation, with the recommendations in the International Society for Applied Ethology guidelines for the use of animals in research, as well as institutional guidelines. The Hungarian “Animal Experiments Scientific and Ethical Committee” approved the experimental procedures under the number: PEI/001/1457-4/2015. The owners provided written consent for their dogs' participation.

### Subjects

#### Study 1

Twenty-six dogs (age ranged from 1 to 18 years old) were included in this study. The sex, breed, and body weight of the animals are presented in Table 1. The relative ages of the animals were calculated as chronological age ^*^ 100/expected lifespan of the breed (33). In the case of mixed-breed dogs, the expected lifespan was calculated by the formula postulated by Greer et al. (34) [version with cm and kg instead of inch and pound were provided by Szabó et al. (35): 13.62 + height_in_cm ^*^ 0.03 - weight_in_kg ^*^ 0.12].

**Table 1:** Dogs included in study 1.

**Breed**	**Sex**	**Body weight (kg)**	**Body height at withers (cm)**	**Chronological age (months)**	**Expected lifespan (months)**	**Relative age (% of expected)**
Border collie	Female	10.7	45	9	147	8
Boxer	Female	20.4	61	14	123	9
Chihuahua	Female	3.6	25	24	149	16
Beagle	Female	7.5	38	48	152	31
Beagle	Female	9.7	38	48	152	31
Beagle	Female	10.6	39	48	152	31
Beagle	Female	7.7	36	48	152	31
German shepherd dog	Female	20.2	70	49	120	40
Gordon setter	Male	37.4	69	46	133	36
Mongrel	Male	15.5	51	45	158	28
Yorkshire terrier	Female	2.1	22	72	152	47
Dobermann	Female	25.1	63	96	126	66
Golden retriever	Female	25.8	58	84	147	57
Mongrel	Male	17.9	45	108	153	70
Vizsla	Male	24.5	60	120	162	74
Border collie	Female	20.4	51	142	147	97
Labrador retriever	Male	36.1	57	153	147	106
Border collie	Female	13.2	49	167	147	114
Labrador retriever	Male	28.4	60	168	147	114
Labrador retriever	Female	25.4	56	161	146	110
Labrador retriever	Male	26.9	59	163	147	114
Dachshund	Female	5	28	204	152	134
Golden retriever	Female	22	56	197	147	138
Mongrel	Female	7.4	45	204	168	121
Small Münsterlander	Female	12.7	47	201	150	136
Mongrel	Male	8.6	35	204	163	125

#### Study 2

Two whole blood samples were taken from 15 border collie dogs (their age ranged from 9 months to 15 years old, Table 2). In this study, no correction for relative age was necessary due to the homogenous breed composition of the sample.

**Table 2:** Border collie dogs included in study 2.

**Age (months)**	**Sex**
9	Female
15	Male
17	Female
17	Female
41	Female
58	Female
48	Male
121	Female
120	Female
125	Male
129	Male
138	Female
144	Male
171	Male
183	Female

### Donation and Sampling

#### Study 1

The animals used in study 1 were donated to the Hungarian Canine Brain and Tissue Bank (CBTB) by their owners, who had previously been informed about the donation possibility through various forums, e.g., social media [(36), submitted]. The animals were euthanized by their veterinarian only for justified medical reasons. Both the owner and the veterinarian gave written consent for the euthanasia and the donation and confirmed that the animal had not had infectious zoonosis prior to euthanasia and had been vaccinated against rabies. After the dogs were euthanized, the bodies were immediately transported to the facility of the CBTB, and dissection was performed by a veterinary anatomist (KC) to obtain tissues within a pre-set 4 h post-mortem limit. After removal from the skull, whole brains were rinsed in phosphate-buffered saline for 5 min, and then cortical samples were removed bilaterally from the area of the frontal lobe. Muscle tissue pieces were obtained from the medial aspect of the musculus temporalis and skin was harvested from the head of the animals. Each tissue piece was cut to be 80–120 mg in mass and was immersed in 1 ml RNAlater. The samples were then incubated at 4°C overnight to enable total percolation of the RNAlater. The next day, the supernatant was removed, and the tissues were frozen at −80 or −20°C for long-term or short-term storage, respectively. Blood was not collected for molecular investigations during standard post-mortem sampling protocols as it was previously found to be highly hemolyzed due to the application of T-61 as euthanasia agent (37).

#### Study 2

Blood was collected from living companion dogs. The date of birth and the breed of the dogs were confirmed based on the dog's pedigree and/or dog passports verified by their veterinarians. Based on the owners' reports, all dogs were healthy and did not take any medication at least 2 weeks before the sampling. One milliliter blood was collected into an EDTA tube and immediately sent to a veterinarian diagnostic laboratory to verify the animals' health status. The blood collection was performed by an experienced veterinarian. Additional 3 ml blood samples were collected into DNA/RNA Blood Collection Tubes (Zymo Research), and were mixed gently, according to the manufacturer's protocol. The blood-filled tubes were stored in a −20°C freezer until further processing.

### RNA Isolation

#### Study 1

Total cellular RNA was isolated from the frontal cortex, temporal muscle, and skin tissue samples stored frozen in RNAlater using TRIzol (Thermo Fisher) and following the manufacturer's protocol. Prior to immersing the tissue pieces in TRIzol, each piece was rinsed in 1 ml of sterile PBS in a new tube and centrifuged for 5 min on 500 g. TRIzol was added to the samples after removal of the PBS. Homogenization of the tissue pieces was carried out by an Ultra-Turrax homogenizer (Ika) under a fume hood to prevent inhalation of TRIzol. After homogenization, the isolation process took place following standard procedures of TRIzol based RNA isolation.

#### Study 2

RNA was isolated from blood samples using the Quick-DNA/RNA Blood Tube Kit (Zymo Research) following the manufacturer's protocol.

The integrity of all isolates was checked by agarose gel electrophoresis. Purity and concentrations were measured by a NanoDrop 1000 device (Thermo Fisher). RNA samples were stored in a −80°C freezer.

### *cDNA* Synthesis and Real-Time PCR

Thousand nanogram of each total RNA was reverse transcribed into cDNA using the Maxima RevertAid cDNA Synthesis Kit (Thermo Fisher). The reactions were performed following the manufacturer's protocol, using random hexamer primers. Prior to downstream applications, the cDNA samples were diluted 10-fold by nuclease-free water and kept at −20°C. For long-term storage, the cDNA samples were placed at −80°C.

Quantitative Real-Time PCR reactions were performed in 10 μl total volume with 3 technical parallels on a LightCycler 96 Instrument (Roche) using the PowerUp SYBRGreen Master Mix (Thermo Fisher) and specifically designed primers for canine *CDKN2A*, Glyceraldehyde-3-Phosphate Dehydrogenase *(GAPDH)*, Ribosomal Protein Lateral Stalk Subunit P0 *(RPLP0)* and Hydroxymethylbilane Synthase *(HMBS)*. *HMBS* was previously reported to be a highly stable transcript across different canine tissues (38, 39), while *GAPDH* was reported to be highly expressed both in brain and muscle tissue in humans (40, 41). Therefore, these two genes seemed appropriate to cover the expected range of expression in the solid tissues. *RPLP0* was chosen instead of *GAPDH* to test blood, as suggested by previous human studies (42). Primers sequences used to detect each transcript were the following: *CDKN2A* forward: 5′- GAGGGCTTCCTGGACACG, reverse: 5′- TCAATTCTTGAAGTCCGGGCT; *GAPDH* forward: 5′-GAGATCCCGCCAACATCAAATGG, reverse: 5′-TACTTCTCATGGTTCACGCCCAT; *HMBS* forward: 5′-ACTCTCTGAAGGACCTGC, reverse: 5′-CTCTTCTCTGGCAAGGTTTC; *RPLP0* forward: 5′- TTCTCGCTTCTTGGAGGGTG, reverse: 5′- CAGGACCCGCTTGTATCCAT.

### Statistical Analysis

Relative gene expression of *CDKN2A* was determined using the delta Ct method, by applying *GAPDH* and *HMBS* as endogenous controls in hard tissues and *RPLP0* together with *HMBS* in blood. Relative age and frontal cortex *CDNK2A* relative expression values were not normally distributed, therefore we used Spearman correlation for investigating the relationship between the variables in SPSS v25. Receiver operating characteristic (ROC) curve analysis was also performed by this software. In the first study, age categories (“young” and “old”) were divided following human conventions: the threshold of being old is often set at 65 years of age in modern societies and the expected lifespan linked to these populations is around 82 years (43). Therefore, dogs were considered “old” when their relate age were above 80% of the expected lifespan.

## Results

### *CDKN2A* Expression in Dog Tissues

*CDKN2A* mRNA was detectable in all tissue types included in this study: brain (frontal cortex), temporal muscle, skin, and blood. In hard tissues (study 1), the relative expression values of *CDKN2A* were highest in the frontal cortex samples and lowest in the skin samples, while muscle showed a mid-level expression in comparison to the same endogenous control genes (*GAPDH* and *HMBS*). Blood samples (study 2) showed an intermediate expression level between the cortical and skin samples based on the *CDKN2A* / *HMBS* ratio.

### The Correlations Between *CDKN2A* Expression and the Animals' Age Were Tissue-Specific

#### Study 1

We found a positive correlation between the relative age of the animals (see Materials and Methods) and *CDKN2A* relative expression in the frontal cortex and muscle tissue (frontal cortex *CDKN2A/GAPDH*: *r* = 0.757, *p* < 0.001; frontal cortex *CDKN2A/HMBS*: *r* = 0.627, *p* = 0.001; muscle *CDKN2A/GAPDH*: *r* = 0.456, *p* = 0.022; muscle *CDKN2A/HMBS*: *r* = 0.435, *p* = 0.030; Table 3). However, no significant correlation was detected in the skin samples. Regarding data from the muscle samples, one 4-year-old dog was an extreme outlier showing a relative expression level ~5 times higher than the average of the other individuals. When the outlier was excluded from the analysis, the level of the correlation between age and *CDKN2A* relative expression increased in the muscle dataset (muscle *CDKN2A/GAPDH*: *r* = 0.544, *p* < 0.001, *CDKN2A/HMBS*: *r* = 0.515, *p* = 0.010), yet still remained much weaker than in the brain tissue of the same animals.

**Table 3:** Spearman's correlations between relative age and *CDKN2A* relative expression values.

		**Relative age**	**Frontal cortex/*GAPDH***	**Frontal cortex/*HMBS***	**Skin/*GAPDH***	**Skin/*HMBS***	**Muscle/*GAPDH***	**Muscle/*HMBS***
Relative age	Correlation Coefficient	1.000	0.757^**^	0.627^**^	0.257	0.148	0.456^*^	0.435^*^
	p (2-tailed)		0.000	0.001	0.236	0.499	0.022	0.030
	*N*	26	26	26	23	23	25	25
Frontal cortex/*GAPDH*	Correlation Coefficient	0.757^**^	1.000	0.902^**^	0.135	−0.164	0.559^**^	0.533^**^
	p (2-tailed)	0.000		0.000	0.538	0.455	0.004	0.006
	*N*	26	26	26	23	23	25	25
Frontal cortex/*HMBS*	Correlation Coefficient	0.627^**^	0.902^**^	1.000	0.073	−0.323	0.624^**^	0.627^**^
	p (2-tailed)	0.001	0.000		0.740	0.133	0.001	0.001
	*N*	26	26	26	23	23	25	25
Skin/*GAPDH*	Correlation Coefficient	0.257	0.135	0.073	1.000	0.398	0.074	0.048
	p (2-tailed)	0.236	0.538	0.740		0.060	0.745	0.832
	N	23	23	23	23	23	22	22
Skin/*HMBS*	Correlation Coefficient	0.148	−0.164	−0.323	0.398	1.000	−0.326	−0.373
	p (2-tailed)	0.499	0.455	0.133	0.060		0.139	0.087
	*N*	23	23	23	23	23	22	22
Muscle /*GAPDH*	Correlation Coefficient	0.456^*^	0.559^**^	0.624^**^	0.074	−0.326	1.000	0.981^**^
	p (2-tailed)	0.022	0.004	0.001	0.745	0.139		0.000
	*N*	25	25	25	22	22	25	25
Muscle/*HMBS*	Correlation Coefficient	0.435^*^	0.533^**^	0.627^**^	0.048	−0.373	0.981^**^	1.000
	p (2-tailed)	0.030	0.006	0.001	0.832	0.087	0.000	
	N	25^*^	25	25	22	22	25	25

* Indicates significant correlations *p < 0.05,

** *p < 0.01*,

*** *p < 0.001*.

*CDKN2A* relative expression values correlated between the frontal cortex and muscle, however the expression in skin did not show such correlations with the other tissue types (Table 3). Neither of the other known variables (weight, height, or sex of the animals) affected the *CDKN2A* relative expression values.

ROC curve analysis was applied to test the capacity of *CDKN2A* relative expression to predict the relative age of the animals in the three tissue types. AUC values exceeded 0.9 in the brain tissue independently of the used endogenous control gene (*CDKN2A*/*GAPDH* AUC = 0.976; *CDKN2A*/*HMBS* AUC = 0.921, *p* < 0.01). Similarly to the correlation results, AUC was smaller, but still significant in the muscle (*CDKN2A*/*GAPDH* AUC = 0.853; *CDKN2A*/*HMBS* AUC = 0.846, *p* < 0.01) and were around the 0.5 random level in the skin tissue.

#### Study 2

In the second study, in which blood samples obtained from border collies were analyzed, the relative expression values of *CDKN2A* also showed a significant, positive correlation with the age of the animals (*CDKN2A*/*RPLP0*: *r* = 0.592, *p* < 0.05; *CDKN2A*/*HMBS*: *r* = 0.539, *p* < 0.05).

ROC curve analysis, however, barely exceeded the 0.5 level (0.695 and 0.781, for *HMBS* and *RPLP0* controls, respectively) in the blood and significance did not reach the *p* < 0.05 values.

## Discussion

We showed that *CKDN2A* expression positively correlated with chronological age in the frontal cortex and temporal muscle tissues, but not in skin tissues of dogs belonging to various breeds. These findings were in accordance with previous reports from humans, where both brain and muscle were found to have highly elevated levels of *CDKN2A* in older people, while an elevation in the skin was moderate and did not reach significance (44, 45). In our study, one outlier among the muscle samples belonged to a dog which had a medical history of epileptic seizures and was euthanized because of a grand mal seizure. The muscle tissue of this dog showed a greatly elevated relative expression of *CDKN2A*. Literature is scarce regarding the effects of seizures on muscle damage (46) and how this would affect the expression of *CDKN2A*. One study has shown that acute muscle damage leads to an increase in the number of senescent cells with elevated *CDKN2A* expression at least in certain muscle-associated cell types (47). In another study, an elevation in the expression of the *p16*^*INK*4*A*^ transcript, a product of the *CDKN2A* gene, was reported in acute kidney injury in young mice (48). Therefore, it is tempting to hypothesize that generalized muscle convulsions could have caused the dramatically elevated *CDKN2A* levels in the muscle tissue of the outlier animal, as no similar elevation was detected in the brain or skin. This hypothesis could also pave the way for investigating *CDKN2A* as a marker of tissue damage. It has recently been suggested by single-cell transcriptomics that the robust, age-related elevation in *CDKN2A* levels may be caused by the increasing ratio of senescent cells with higher damage/mutational load and not by an intrinsic aging program that generally upregulates its expression in all cells (49). The increased individual variability seen in muscle and skin may have contributed to the decrease/lack of correlation between age and *CDKN2A* gene expression in these tissues. As another possible confounder, the expression stability of the applied housekeeping genes should also be considered. Although the choice of *GAPDH* and *HMBS* for solid tissues was based on previous literature data indicating these as generally stable transcripts, no previous data was available regarding the possible age-related changes of these genes' expression in dog tissues. In the case of *HMBS*, we detected a marginal, negative correlation between expression and dogs' age in the muscle tissue (*r* = −0.417, *p* < 0.05), indicating that this gene was not an optimal control to assess age-related gene expression changes. This finding underlines why it is advisable to include more than one control gene in an RT-qPCR experiment (50).

Importantly, the tissue-specific correlations we detected in our dog population were concordant with previous findings reported from humans (44, 51). Although none of these studies compared frontal cortex, skin and muscle in a similar setting as reported herein, pairwise comparisons between muscle and skin (44) or brain and skin (52) showed similar patterns, reporting no significant changes in *CDKN2A* expression in skin, only in the other tissues. Indications for similar tissue specificity were also reported in mice, together with a generally huge variance in the number of genes to show age-related changes in different tissues (45, 53). This indicates that age-related gene expression changes commonly exhibit distinct tissue specific patterns. Furthermore, individual differences were reported in both mice and humans, with extreme outliers occurring in several datasets (53, 54). This phenomenon was also seen in humans when the same tissues from different body parts (e.g., skin from sun-exposed vs. protected areas) were compared (54). Altogether, this suggests that age-related gene expression changes can vary widely between samples, depending on species, genetic background, tissue type, and environmental factors. In this regard, our findings could be an important addition to *CDKN2A* biomarker research, because we used a mixed population of dogs in study 1, including several breeds and mongrels in the analysis. Therefore, we demonstrated that *CDKN2A* could be a powerful tool for following age-related changes in certain tissues of dogs, independently of individual genetic background.

In humans the most important feature of *CDKN2A* as a biomarker of aging is its availability in the blood and the validated correlations between its blood expression levels and age/cellular damage or senescence in other tissues (26, 28–30). In order to gain insight into the expression changes of *CDKN2A* in the blood of dogs, we investigated a second cohort of animals. Due to considerations related to a different study, this cohort included only animals from the same breed (border collie). Therefore, the results of study 2 are not indicative of the expression variance of *CDKN2A* in a multi-breed dog population. Comparisons between different breeds will be required, to test if blood sample data for *CDKN2A* expression behaves similarly stable across animals of different genetic backgrounds. Also, the correlation between *CDKN2A* expression and age was weaker in the blood than seen in the brain tissue in study 1, and the ROC curve analysis did not indicate a good predictive capacity in this sample population. Importantly, one of the main reasons for this could be the large variability seen in individual expression values in the blood, coupled with the limited sample number of 5 old vs. 5 young animals. Human studies suggested that reliable gene expression detection might be challenging from whole blood samples, which are a mixture of different cell types (55). Therefore, further optimization of the method used to analyze blood, including the separation of white blood cells (“buffy coat”), may yield more consistent results regarding the link between *CDKN2A* levels and biological age. In humans, T lymphocytes were reported to show the most robust *CKDN2A* expression change with aging (26).

A main limitation of our study is the lack of solid tissue and blood samples from the same animals. The reason was methodological: all animals were euthanized by chemical agents, which causes severe hemolysis (37, 56). We found no data about the comparability of hemolyzed blood samples with normal samples regarding mRNA gene expression analyses, while in regard to miRNA-s it was reported that hemolysis could modify the results (57). Therefore, the use of hemolyzed blood samples was not considered appropriate for our study aims. Another major limitation is the limited size and non-continuous composition of the sample population in study 2. The animals participating in this study were recruited for other ongoing study goals, where a young-old age composition was required. For practical reasons – e.g., the limited mobility of both owners and personnel due to the COVID outbreak -, the number of involved animals could not be increased to include dogs with intermediate ages.

Altogether, our findings about the robust age-related increase in *CDKN2A* mRNA levels in some dog tissues propose an evolutionary conserved aging pathway, which could be applied as a reliable aging marker in dogs, increasing the number of currently available blood-borne biomarkers in this species (11, 12, 16, 58, 59). Previous studies in humans suggest that the number of simultaneously assessed markers could dramatically increase the efficiency of disease categorization and prediction, especially in the case of various forms of dementia (60–63). Importantly, *CDKN2A* has already been implicated as a regulator (64, 65) and biomarker (66) of human Alzheimer's disease (AD), however, its exact link to disease occurrence and progression is still unclear (67).

Since dogs have been proposed as natural models of human AD (68) and live shorter lives than humans, future longitudinal studies conducted in dogs may help researchers to characterize the links between blood *CKDN2A* levels and age-related brain pathologies more precisely. Although the validation of blood *CDKN2A* level as a reliable marker of aging will require further experiments, our preliminary findings indicate that *CDKN2A* could be a promising predictor of biological aging, and possibly age-related pathologies in the dog.

## Data Availability Statement

The raw data supporting the conclusions of this article will be made available by the authors, without undue reservation.

## Ethics Statement

The animal study was reviewed and approved by Hungarian Animal Experiments Scientific and Ethical Committee. Written informed consent was obtained from the owners for the participation of their animals in this study.

## Author Contributions

SS designed study plan, performed laboratory work, did statistical analyses, and wrote the manuscript. KT performed laboratory work. KC provided samples for the laboratory work and contributed to writing. BE provided laboratory equipment and materials and contributed to writing. EK contributed to writing, did statistical analyses, and provided funding. All authors contributed to the article and approved the submitted version.

## Conflict of Interest

The authors declare that the research was conducted in the absence of any commercial or financial relationships that could be construed as a potential conflict of interest.

## References

[1] Gilmore KM Greer KA. Why is the dog an ideal model for aging research? Exp Gerontol. (2015) 71:14–20. 10.1016/j.exger.2015.08.008 26325590

[2] Kaeberlein M Creevy KE Promislow DEL. The dog aging project: translational geroscience in companion animals. Mamm Genome. (2016) 27:279–88. 10.1007/s00335-016-9638-7 27143112 PMC4936929

[3] Mazzatenta A Carluccio A Robbe D Di Giulio C Cellerino A. The companion dog as a unique translational model for aging. Semin Cell Dev Biol. (2017) 70:141–53. 10.1016/j.semcdb.2017.08.024 28803893

[4] Hoffman JM Creevy KE Franks A O'Neill DG Promislow DEL. The companion dog as a model for human aging and mortality. Aging Cell. (2018) 17:e12737. 10.1111/acel.12737 29457329 PMC5946068

[5] Kennedy BK Berger SL Brunet A Campisi J Cuervo AM Epel ES. Geroscience: linking aging to chronic disease. Cell. (2014) 159:709–13. 10.1016/j.cell.2014.10.039 25417146 PMC4852871

[6] Prospective Studies Collaboration Lewington S Whitlock G Clarke R Sherliker P Emberson J. Blood cholesterol and vascular mortality by age, sex, and blood pressure: a meta-analysis of individual data from 61 prospective studies with 55 000 vascular deaths. Lancet. (2007) 370:1829–39. 10.1016/S0140-6736(07)61778-4 18061058

[7] Lewington S Clarke R Qizilbash N Peto R Collins R Prospective Studies Collaboration. Age-specific relevance of usual blood pressure to vascular mortality: a meta-analysis of individual data for one million adults in 61 prospective studies. Lancet. (2002) 360:1903–13. 10.1016/S0140-6736(02)11911-8 12493255

[8] Horvath S Raj K. DNA methylation-based biomarkers and the epigenetic clock theory of ageing. Nat Rev Genet. (2018) 19:371–84. 10.1038/s41576-018-0004-3 29643443

[9] Levine ME Lu AT Quach A Chen BH Assimes TL Bandinelli S. An epigenetic biomarker of aging for lifespan and healthspan. Aging. (2018) 10:573–91. 10.18632/aging.101414 29676998 PMC5940111

[10] Mozhui K Pandey AK. Conserved effect of aging on DNA methylation and association with EZH2 polycomb protein in mice and humans. Mech Ageing Dev. (2017) 162:27–37. 10.1016/j.mad.2017.02.006 28249716 PMC5411177

[11] Thompson MJ von Holdt B Horvath S Pellegrini M. An epigenetic aging clock for dogs and wolves. Aging. (2017) 9:1055–68. 10.18632/aging.101211 28373601 PMC5391218

[12] Wang T Ma J Hogan AN Fong S Licon K Tsui B. Quantitative translation of dog-to-human aging by conserved remodeling of the DNA methylome. Cell Syst. (2020) 11:176–85.e6. 10.1016/j.cels.2020.06.006 32619550 PMC7484147

[13] Sebastiani P Thyagarajan B Sun F Schupf N Newman AB Montano M. Biomarker signatures of aging. Aging Cell. (2017) 16:329–38. 10.1111/acel.12557 28058805 PMC5334528

[14] Kudryashova KS Burka K Kulaga AY Vorobyeva NS Kennedy BK. Aging biomarkers: from functional tests to multi-omics approaches. Proteomics. (2020) 20:1900408. 10.1002/pmic.201900408 32084299

[15] Justice JN Ferrucci L Newman AB Aroda VR Bahnson JL Divers J. A framework for selection of blood-based biomarkers for geroscience-guided clinical trials: report from the TAME Biomarkers Workgroup. GeroScience. (2018) 40:419–36. 10.1007/s11357-018-0042-y 30151729 PMC6294728

[16] Panek WK Gruen ME Murdoch DM Marek RD Stachel AF Mowat FM. Plasma neurofilament light chain as a translational biomarker of aging and neurodegeneration in dogs. Mol Neurobiol. (2020) 57:3143–9. 10.1007/s12035-020-01951-0 32472519 PMC7529326

[17] Melk A Schmidt BMW Takeuchi O Sawitzki B Rayner DC Halloran PF. Expression of p16INK4a and other cell cycle regulator and senescence associated genes in aging human kidney. Kidney Int. (2004) 65:510–20. 10.1111/j.1523-1755.2004.00438.x 14717921

[18] Nielsen GP Stemmer-Rachamimov AO Shaw J Roy JE Koh J Louis DN. Immunohistochemical survey of p16INK4A expression in normal human adult and infant tissues. Lab Invest. (1999) 79:1137–43. 10496532

[19] Zindy F Quelle DE Roussel MF Sherr CJ. Expression of the p16INK4a tumor suppressor versus other INK4 family members during mouse development and aging. Oncogene. (1997) 15:203–11. 10.1038/sj.onc.1201178 9244355

[20] Foulkes WD Flanders TY Pollock PM Hayward NK. The *CDKN2A* (p16) gene and human cancer. Mol Med. (1997) 3:5–20. 10.1007/BF03401664 9132280 PMC2230107

[21] Sharpless NE. Ink4a/Arf links senescence and aging. Exp Gerontol. (2004) 39:1751–9. 10.1016/j.exger.2004.06.025 15582292

[22] Shiels PG. *CDKN2A* might be better than telomere length in determining individual health status. BMJ. (2012) 344:e1415. 10.1136/bmj.e1415 22395926

[23] Gingell-Littlejohn M McGuinness D McGlynn LM Kingsmore D Stevenson KS Koppelstaetter C. Pre-transplant *CDKN2A* expression in kidney biopsies predicts renal function and is a future component of donor scoring criteria. PLoS ONE. (2013) 8:e68133. 10.1371/journal.pone.0068133 23861858 PMC3701657

[24] Koppelstaetter C Schratzberger G Perco P Hofer J Mark W Öllinger R. Markers of cellular senescence in zero hour biopsies predict outcome in renal transplantation. Aging Cell. (2008) 7:491–7. 10.1111/j.1474-9726.2008.00398.x 18462273

[25] Krishnamurthy J Torrice C Ramsey MR Kovalev GI Al-Regaiey K Su L. Ink4a/Arf expression is a biomarker of aging. J Clin Invest. (2004) 114:1299–307. 10.1172/JCI22475 15520862 PMC524230

[26] Liu Y Sanoff HK Cho H Burd CE Torrice C Ibrahim JG. Expression of *p16* ^INK4a^ in peripheral blood T-cells is a biomarker of human aging. Aging Cell. (2009) 8:439–48. 10.1111/j.1474-9726.2009.00489.x 19485966 PMC2752333

[27] Vandenberk B Brouwers B Hatse S Wildiers H. P16INK4a: A central player in cellular senescence and a promising aging biomarker in elderly cancer patients. J Geriatr Oncol. (2011) 2:259–69. 10.1016/j.jgo.2011.08.004

[28] Robinson MW McGuinness D Swann R Barclay S Mills PR Patel AH. Non cell autonomous upregulation of CDKN2 transcription linked to progression of chronic hepatitis C disease. Aging Cell. (2013) 12:1141–3. 10.1111/acel.12125 23931242

[29] Asghar M Yman V Homann MV Sondén K Hammar U Hasselquist D. Cellular aging dynamics after acute malaria infection: a 12-month longitudinal study. Aging Cell. (2018) 17:e12702. 10.1111/acel.12702 29143441 PMC5771395

[30] Banerjee J Dhas Y Mishra N. Middle-aged indians with type 2 diabetes are at higher risk of biological ageing with special reference to serum *CDKN2A*. J Diabetes Res. (2020) 2020:10. 10.1155/2020/7569259 32280716 PMC7128035

[31] Sorrentino JA Sanoff HK Sharpless NE. Defining the toxicology of aging. Trends Mol Med. (2014) 20:375–84. 10.1016/j.molmed.2014.04.004 24880613 PMC4082749

[32] Sándor S Kubinyi E. Genetic pathways of aging and their relevance in the dog as a natural model of human aging. Front Genet. (2019) 10:948. 10.3389/fgene.2019.00948 31681409 PMC6813227

[33] Adams VJ Evans KM Sampson J Wood JLN. Methods and mortality results of a health survey of purebred dogs in the UK. J Small Anim Pract. (2010) 51:512–24. 10.1111/j.1748-5827.2010.00974.x 21029096

[34] Greer KA Canterberry SC Murphy KE. Statistical analysis regarding the effects of height and weight on life span of the domestic dog. Res Vet Sci. (2007) 82:208–14. 10.1016/j.rvsc.2006.06.005 16919689

[35] Szabó D Gee NR Miklósi Á. Natural or pathologic? Discrepancies in the study of behavioral and cognitive signs in aging family dogs. J Vet Behav. (2016) 11:86–98. 10.1016/j.jveb.2015.08.003

[36] Sándor S Czeibert K Salamon A Kubinyi E. Man's best friend in life and death: scientific perspectives and challenges of dog brain banking (unpublished manuscript). 10.1007/s11357-021-00373-7 PMC8492856 33970413

[37] Prien T Traber DL Linares HA Davenport SL. Haemolysis and artifactual lung damage induced by an euthanasia agent. Lab Anim. (1988) 22:170–2. 10.1258/002367788780864420 3392952

[38] Park S-J Huh J-W Kim Y-H Lee S-R Kim S-H Kim S-U. Selection of internal reference genes for normalization of quantitative reverse transcription polymerase chain reaction (qRT-PCR) analysis in the canine brain and other organs. Mol Biotechnol. (2013) 54:47–57. 10.1007/s12033-012-9543-6 22531949

[39] Stassen QEM Riemers FM Reijmerink H Leegwater PAJ Penning LC. Reference genes for reverse transcription quantitative PCR in canine brain tissue. BMC Res Notes. (2015) 8:761. 10.1186/s13104-015-1628-4 26654363 PMC4673830

[40] Radonić A Thulke S Mackay IM Landt O Siegert W Nitsche A. Guideline to reference gene selection for quantitative real-time PCR. Biochem Biophys Res Commun. (2004) 313:856–62. 10.1016/j.bbrc.2003.11.177 14706621

[41] Barber RD Harmer DW Coleman RA Clark BJ. GAPDH as a housekeeping gene: analysis of GAPDH mRNA expression in a panel of 72 human tissues. Physiol Genomics. (2005) 21:389–95. 10.1152/physiolgenomics.00025.2005 15769908

[42] Dheda K Huggett JF Chang JS Kim LU Bustin SA Johnson MA. The implications of using an inappropriate reference gene for real-time reverse transcription PCR data normalization. Anal Biochem. (2005) 344:141–3. 10.1016/j.ab.2005.05.022 16054107

[43] United Nations Department of Economic and Social Affairs Population Division. World Population Ageing 2019: Highlights (ST/ESA/SER.A/430), New York, NY, (2019).

[44] Ardlie KG DeLuca DS Segrè AV Sullivan TJ Young TR Gelfand ET. The genotype-tissue expression (GTEx) pilot analysis: multitissue gene regulation in humans. Science (80-). (2015) 348:648–60. 10.1126/science.1262110 25954001 PMC4547484

[45] Hudgins AD Tazearslan C Tare A Zhu Y Huffman D Suh Y. Age- and tissue-specific expression of senescence biomarkers in mice. Front Genet. (2018) 9:59. 10.3389/fgene.2018.00059 29527222 PMC5829053

[46] Sutter R Dittrich T Semmlack S Rüegg S Marsch S Kaplan PW. Acute systemic complications of convulsive status epilepticus — a systematic review. Crit Care Med. (2018) 46:138–45. 10.1097/CCM.0000000000002843 29099419

[47] Saito Y Chikenji TS Matsumura T Nakano M Fujimiya M. Exercise enhances skeletal muscle regeneration by promoting senescence in fibro-adipogenic progenitors. Nat Commun. (2020) 11:889. 10.1038/s41467-020-14734-x 32060352 PMC7021787

[48] Gu X Peng CY Lin SY Qin ZY Liang JL Chen HJ. P16INK4a played a critical role in exacerbating acute tubular necrosis in acute kidney injury. Am J Transl Res. (2019) 11:3850–61. 31312394 PMC6614612

[49] Enge M Arda HE Mignardi M Beausang J Bottino R Kim SK. Single-cell analysis of human pancreas reveals transcriptional signatures of aging and somatic mutation patterns. Cell. (2017) 171:321–30.e14. 10.1016/j.cell.2017.09.004 28965763 PMC6047899

[50] Vandesompele J De Preter K Pattyn F Poppe B Van Roy N De Paepe A. Accurate normalization of real-time quantitative RT-PCR data by geometric averaging of multiple internal control genes. Genome Biol. (2002) 3:research0034.1. 10.1186/gb-2002-3-7-research0034 12184808 PMC126239

[51] Glass D Viñuela A Davies MN Ramasamy A Parts L Knowles D. Gene expression changes with age in skin , adipose tissue , blood and brain. Genome Biol. (2013) 14:R75. 10.1186/gb-2013-14-7-r75 23889843 PMC4054017

[52] Shavlakadze T Morris M Fang J Wang SX Zhu J Zhou W. Age-related gene expression signature in rats demonstrate early, late, and linear transcriptional changes from multiple tissues. Cell Rep. (2019) 28:3263–73.e3. 10.1016/j.celrep.2019.08.043 31533046

[53] Zahn JM Poosala S Owen AB Ingram DK Lustig A Carter A. AGEMAP: a gene expression database for aging in mice. PLoS Genet. (2007) 3:e201. 10.1371/journal.pgen.0030201 18081424 PMC2098796

[54] Yang J Huang T Petralia F Long Q Zhang B Argmann C. Synchronized age-related gene expression changes across multiple tissues in human and the link to complex diseases. Sci Rep. (2015) 5:1–16. 10.1038/srep15145 26477495 PMC4609956

[55] Fan H Hegde P. The transcriptome in blood: challenges and solutions for robust expression profiling. Curr Mol Med. (2005) 5:3–10. 10.2174/1566524053152861 15720265

[56] Doughty MJ Stuart D. Quantification of the hemolysis associated with use of T-61(R) as a euthanasia agent in rabbits - A comparison with Euthanyl(R). (pentobarbital sodium) and the impact on serum hexosaminidase measurements. Can J Physiol Pharmacol. (1995) 73:1274−80. 10.1139/y95-179 8748976

[57] Kirschner MB Edelman JJB Kao SC-H Vallely MP van Zandwijk N Reid G. The impact of hemolysis on cell-free microRNA biomarkers. Front Genet. (2013) 4:94. 10.3389/fgene.2013.00094 23745127 PMC3663194

[58] McKevitt TP Nasir L Devlin P Argyle DJ. Telomere lengths in dogs decrease with increasing donor age. J Nutr. (2002) 132:1604S−6S. 10.1093/jn/132.6.1604S 12042469

[59] Fick LJ Fick GH Li Z Cao E Bao B Heffelfinger D. Telomere length correlates with life span of dog breeds. Cell Rep. (2012) 2:1530–6. 10.1016/j.celrep.2012.11.021 23260664

[60] Frölich L Peters O Lewczuk P Gruber O Teipel SJ Gertz HJ. Incremental value of biomarker combinations to predict progression of mild cognitive impairment to Alzheimer's dementia. Alzheimer's Res Ther. (2017) 9:1–15. 10.1186/s13195-017-0301-7 29017593 PMC5634868

[61] Meeter LH Donker Kaat L Rohrer JD van Swieten JC. Imaging and fluid biomarkers in frontotemporal dementia. Nat Rev Neurol. (2017) 13:406–19. 10.1038/nrneurol.2017.75 28621768

[62] Fransquet PD Ryan J. The current status of blood epigenetic biomarkers for dementia. Crit Rev Clin Lab Sci. (2019) 56:435–57. 10.1080/10408363.2019.1639129 31328605

[63] Parnetti L Paciotti S Farotti L Bellomo G Sepe FN Eusebi P. Parkinson's and Lewy body dementia CSF biomarkers. Clin Chim Acta. (2019) 495:318–25. 10.1016/j.cca.2019.04.078 31051162

[64] Züchner S Gilbert JR Martin ER Leon-Guerrero CR Xu P-T Browning C. Linkage and association study of late-onset Alzheimer disease families linked to 9p21.3. Ann Hum Genet. (2008) 72:725–31. 10.1111/j.1469-1809.2008.00474.x 18761660 PMC2821735

[65] Emanuele E Lista S Ghidoni R Binetti G Cereda C Benussi L. Chromosome 9p21.3 genotype is associated with vascular dementia and Alzheimer's disease. Neurobiol Aging. (2011) 32:1231–5. 10.1016/j.neurobiolaging.2009.07.003 19664850

[66] Antonell A Lladó A Sánchez-Valle R Sanfeliu C Casserras T Rami L. Altered blood gene expression of tumor-related genes (PRKCB, BECN1, and *CDKN2A*) in Alzheimer's disease. Mol Neurobiol. (2016) 53:5902–11. 10.1007/s12035-015-9483-9 26510741

[67] Tedde A Piaceri I Bagnoli S Lucenteforte E Ueberham U Arendt T. Association study of genetic variants in *CDKN2A*/CDKN2B genes/loci with late-onset alzheimer's disease. Int J Alzheimers Dis. (2011) 2011:374631. 10.4061/2011/374631 21559192 PMC3090043

[68] Schütt T Helboe L Pedersen LØ Waldemar G Berendt M Pedersen JT. Dogs with cognitive dysfunction as a spontaneous model for early alzheimer's disease: a translational study of neuropathological and inflammatory markers. J Alzheimers Dis. (2016) 52:433–49. 10.3233/JAD-151085 27003213

